# Tumor morphology on CT radiomics is largely driven by the local anatomical environment, not the primary tumor type

**DOI:** 10.1186/s41747-026-00691-5

**Published:** 2026-03-12

**Authors:** Sajjad Rostami, Corentin Guérendel, Marleen Soliman, Hannah W. Stutterheim, Olga Maxouri, Diana Ivonne Rodríguez Sánchez, Stephan Ursprung, Nino Boveradze, George Agrotis, Kalina Chupetlovska, Francesca Castagnoli, Federica Landolfi, Eun Kyoung Hong, Andrea Delli Pizzi, Nicolo Gennaro, Mohamed A. Abdelatty, Warissara Jutidamrongphan, Liliana Petrychenko, Peter Matkulcik, Alba Salgado-Parente, Francesco Marcello Arico, Sean Benson, Petur Snaebjornsson, Zuhir Bodalal, Regina G. H. Beets-Tan

**Affiliations:** 1 Department of Radiology, The Netherlands Cancer Institute, Amsterdam, The Netherlands; 2 GROW Research Institute for Oncology and Reproduction, Maastricht University, Maastricht, The Netherlands; 3 Department of Diagnostic and Interventional Radiology, Tübingen University Hospital, Karls-Eberhardt University, Tübingen, Germany; 4 Department of Radiology, American Hospital Tbilisi, Tbilisi, Georgia; 5 Department of Radiology, Royal Marsden Hospital, London, UK; 6 Division of Radiotherapy and Imaging, The Institute of Cancer Research, London, UK; 7 Radiology Unit, Sant’Andrea Hospital, Sapienza University of Rome, Rome, Italy; 8 Department of Radiology, Stanford University, Palo Alto, CA USA; 9 Department of Innovative Technologies in Medicine & Dentistry, G. d’Annunzio University of Chieti-Pescara, Chieti, Italy; 10 Institute for Advanced Biomedical Technologies, G. d’Annunzio University of Chieti-Pescara, Chieti, Italy; 11 Feinberg School of Medicine, Northwestern University, Chicago, IL USA; 12 Clinic of Radiology, Imaging Institute of Southern Switzerland (IIMSI), Ente Ospedaliero Cantonale (EOC), Lugano, Switzerland; 13 Department of Radiology, Kasr Al-Ainy Hospital, Cairo University, Cairo, Egypt; 14 Department of Nuclear Medicine, Inselspital, Bern University Hospital, University of Bern, Bern, Switzerland; 15 Department of Radiology and Nuclear Medicine, University Hospital Brno, Brno, Czechia; 16 Department of Radiology, Ramón y Cajal University Hospital, Madrid, Spain; 17 Diagnostic and Interventional Radiology Unit, BIOMORF Department, University Hospital “Policlinico G. Martino”, Messina, Italy; 18 Department of Cardiology, Amsterdam University Medical Center, University of Amsterdam, Amsterdam, The Netherlands; 19 Department of Pathology, The Netherlands Cancer Institute, Amsterdam, The Netherlands; 20 Faculty of Medicine, University of Iceland, Reykjavik, Iceland; 21 The Netherlands Cancer Institute, Amsterdam, The Netherlands; 22 Faculty of Health Sciences, University of Southern Denmark, Odense, Denmark; 23 Maastricht Radiation Oncology, Maastricht, The Netherlands

**Keywords:** Biomarkers, Neoplasms, Radiomics, Tomography (x-ray computed), Tumor microenvironment

## Abstract

**Objective:**

Radiogenomics promises noninvasive tumor profiling; however, the extent to which imaging morphology reflects tumor lineage *versus* host-organ milieu remains unclear. This study aimed to quantify the relative influence of tumor type and anatomical environment on contrast-enhanced computed tomography (CT) radiomic phenotypes.

**Materials and methods:**

A discovery cohort of 1,598 patients (10,485 lesions) and an external validation cohort of 2,440 patients (6,597 lesions) underwent portal-venous-phase CT. After manual segmentation, lesion-level radiomic features were standardized and embedded using *t*-distributed stochastic neighbor embedding. Bayesian-optimized agglomerative clustering defined morphology-based groups. Concordance with the primary tumor site (lineage) and anatomical environment was quantified using bootstrapped adjusted Rand indices (ARI); the silhouette score assessed clustering quality. Feature-class (shape, intensity, texture) and mask-erosion experiments probed mechanistic drivers.

**Results:**

Six morphological clusters were identified in the discovery set (silhouette = 0.44). Morphology aligned more strongly with environment (mean ARI = 0.37) but poorly with lineage (mean ARI = 0.04; *p* < 0.010); this pattern held externally. In solid organ metastases, environment dominance was even stronger (mean ARI = 0.60 *versus* 0.05; *p* < 0.010). Intensity and texture drove the morphological association with anatomical environment (ARI = 0.64–0.56) more than shape (ARI = 0.06). When the periphery of the tumor was eroded, the same patterns were observed, implicating the tumor core.

**Conclusion:**

Across organs and tumor types, tumor morphological phenotype on CT imaging is largely driven by a host tissue-related environmental “imprint” rather than the primary tumor site.

**Relevance statement:**

Context-aware modeling is essential for reliable radiomic biomarkers and could motivate a two-step AI pipeline that first identifies the organ habitat and refines lineage-specific predictions.

**Key Points:**

- In a large, multicenter cohort, tumors exhibited distinct morphological clustering.
- These clusters did not align with primary tumor sites (ARI = 0.04).
- Stronger associations emerged between morphological clusters and the local anatomical environment (ARI = 0.37).
- Stratification by lesion type revealed even stronger associations between local anatomical context and solid organ metastases (ARI = 0.60).

**Graphical Abstract:**

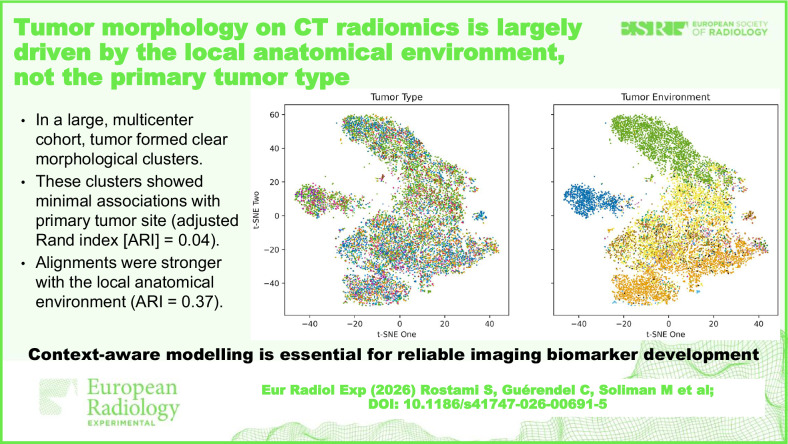

## Background

Precision oncology seeks to match the right treatment to the right patient by accounting for the biological forces that drive tumor behavior, treatment response, or disease progression. A detailed understanding of a tumor’s biological profile allows clinicians to make more informed decisions about prognosis, therapeutic strategies, and monitoring, ultimately improving patient outcomes [1]. Traditionally, such biological insights are obtained through tissue biopsies or other specimen-based approaches. While clinically valuable, biopsies are invasive, carry procedural risks, and can be stressful or painful for patients [2, 3]. Moreover, they often sample only a small portion of the tumor, failing to capture its full heterogeneity [4]. Some tumors are also located in anatomically challenging or unsafe regions, limiting the feasibility of repeated tissue sampling. These biological and practical limitations have sparked interest in noninvasive, image-based strategies that can interrogate all lesions across multiple time points without increasing patient risk.

Radiogenomics has emerged as one such strategy, offering a noninvasive approach to infer tumor biology through quantitative analysis of imaging features [5, 6]. By bridging genomics and radiological imaging, radiogenomics seeks to uncover links between tumor morphology, its structural and visual appearance, and underlying molecular characteristics [7]. Recent prospective work has shown that computed tomography (CT)-radiomic signatures can noninvasively predict actionable driver mutations in non-small cell lung cancer, enabling earlier initiation of targeted therapy, and potentially sparing patients a repeat biopsy [8]. Complementary studies demonstrate that radiomics can also capture hallmarks of the tumor microenvironment, such as hypoxia, immune infiltration, and stromal composition, that modulate response to immunotherapy [9–11]. Notably, radiomics models have also shown promise in distinguishing liver metastases from primary liver cancers using (multiphasic) CT, pointing to the potential of morphology-aware artificial intelligence (AI) in evaluating liver-dominant cancers of unknown primary [12–14].

Despite its promise, the field of radiogenomics remains in its infancy. Many existing studies are preliminary, based on small datasets with limited external validation, which constrains their generalizability [15, 16]. Moreover, most focus on predicting biological endpoints, such as genetic mutations or molecular subtypes, from imaging data [17, 18], while often overlooking contextual factors that may also influence the tumors’ morphological phenotype. A deeper appreciation of the bidirectional relationship between tumor morphology and its underlying biology could substantially enhance the clinical utility and explainability of radiogenomics.

Taken together, these points highlight the need to disentangle the imaging traits shaped by intrinsic tumor biology from those influenced by the host tissue environment. In this study, we investigated how two key biological aspects, primary tumor site and host tissue environment, contribute to tumor morphology. Using high-dimensional radiomics, we profiled lesions from two large multi-cancer cohorts and generated morphological “fingerprints” from contrast-enhanced CT scans. By directly comparing these two biological factors, our study aims to clarify the extent to which host tissue context influences tumor morphology, insights that could refine radiogenomic biomarker development.

## Materials and methods

### Study population and ethical approval

This study included two cohorts: a discovery cohort and an external validation cohort. The discovery cohort retrospectively comprised consecutive cancer patients treated at the Netherlands Cancer Institute, from 2016 to 2020, who underwent biopsy followed by molecular profiling. We queried the institutional Picture Archiving and Communication System (PACS) to identify baseline contrast-enhanced CT scans suitable for medical image analysis. A team of radiologists assessed the resulting images. Exclusion criteria were as follows: multiple synchronous primary tumors, lack of a histologically confirmed diagnosis, poor image quality, slice thickness greater than 5 mm, or no visible (or segmentable) lesions on baseline imaging (Supplementary Fig. S1).

The validation cohort was assembled from 47 publicly available cancer imaging datasets (Supplementary Table S1) [19–60]. Contrast-enhanced CT scans were evaluated for image quality and the presence of tumors. The same exclusion criteria as the discovery cohort were applied.

Ethical approval for this study was granted by the Institutional Review Board of the Netherlands Cancer Institute (IRBdm19-147). The requirement for project-specific informed consent was waived due to the retrospective nature of the analysis. This project adhered to the guidelines listed in the CheckList for EvaluAtion of Radiomics research (CLEAR) criteria [61, 62].

### Image acquisition and retrieval

Scans for the discovery cohort were acquired during patients’ routine clinical workflow and were retrieved from our institutional PACS. For the validation cohort, images were downloaded from publicly accessible repositories (namely, The Cancer Imaging Archive [63], Medical Segmentation Decathlon [26], and WORC [20] databases). In both cohorts, scans were acquired on Philips Healthcare, Siemens Healthineers, GE Healthcare, or Canon Medical Systems scanners with 16–320 channels. CT scans were reconstructed to a slice thickness of 1–5 mm. Patients were administered an iodine-based contrast agent intravenously (90–130 mL of Omnipaque at 300 mg/mL, based on body weight) for the discovery cohort. Portal venous phase scans were acquired 70 s after contrast administration and were used in our image analysis. The portal venous phase was selected for segmentation due to its standardized use in oncologic imaging and assessment. The in-plane resolution was consistent across the two imaging cohorts, indicating comparable diagnostic quality. Both cohorts had a median x and y pixel spacing of 0.8 mm (interquartile range 0.7–0.8 mm). All image and segmentation files were stored according to the Nearly Raw Raster Data‒NRRD standard. Supplementary Table S2 provides detailed information on the image acquisition parameters for both cohorts.

### Image segmentation and feature extraction

Tumor delineations were performed (using 3D Slicer v5.4 [64]) on axial slices by two independent teams, each comprising six to seven radiologists: one team for the discovery set (N.B., F.C., F.L., E.K.H., A.D.P., N.G.; 4–8 years of experience) and another for the validation set (F.M.A., G.A., K.C., M.A., W.J., P.M., A.S.P.; 4–8 years of experience). Tumor delineation was performed with reference to all relevant imaging information available in PACS. Two radiologists (K.C., S.U.; 5–7 years of experience) cross-verified and aligned the segmentations for uniformity within their cohort. Readers segmented all visible tumors on all slices, with a maximum of ten lesions per organ. The minimum lesion size for segmentation was set at a short axis ≥ 10 mm. For each segmented lesion, detailed anatomical information was recorded by a radiologist (S.U.), specifying whether it was a primary tumor or metastasis, as well as its anatomical location. Metastatic lesions were further grouped as: (1) lymph node metastases or (2) solid organ metastases, encompassing all extranodal sites.

Subsequently, for each lesion, 2,016 radiomic features were extracted using PyRadiomics (v3.1.0a1) [65] in accordance with Image Biomarker Standardization Initiative standards [66]. Prior to feature extraction, images were resampled to 1 mm^3^ isotropic voxels using B-spline interpolation. The extracted features included two- and three-dimensional shape features, intensity (first-order) statistics, and texture features, such as gray level co-occurrence matrix—GLCM, gray level run length matrix—GLRLM, gray level size zone matrix—GLSZM, neighboring gray tone difference matrix—NGTDM, and gray level dependence matrix—GLDM. Collectively, these features encapsulate the morphological phenotype of a lesion. An in-depth description of the extracted features can be found in Griethuysen et al [65]. Detailed parameters for the feature extraction are available at Supplementary Methods S1.

### Study design

To explore and visualize morphological variations, we applied *t*-distributed stochastic neighbor embedding (*t*-SNE). This nonlinear, unsupervised dimensionality reduction technique preserves local data structures in high-dimensional spaces [67]. Before generating the *t-*SNE embeddings, radiomic features were standardized to their mean and standard deviation, with imputation (using median) performed in cases where missing values might be encountered. Details of the hyperparameters used in the *t*-SNE process can be found in Supplementary Table S3. The resulting two-dimensional embeddings were visualized as scatter plots, with each point corresponding to an individual lesion; points closer together indicate greater morphological similarity.

Morphological phenotypes were first explored via the t-SNE scatter plots for potential similarities among the tumors. Agglomerative clustering was then applied to these embeddings to quantitatively identify morphologically similar subgroups [68]. Clustering hyperparameters were optimized using Bayesian optimization (Supplementary Table S4). Clustering was also performed using the raw radiomic feature vectors, focusing specifically on metastatic lesions in solid organs for comparison to *t*-SNE embeddings.

Two biologically relevant endpoints were examined in this study, namely *tumor type* (defined as the site of the primary tumor) and tumor environment (defined as the anatomical location of each particular lesion). In order to have sufficient counts per class, we performed grouping of certain anatomical sites together on a systems basis (*e.g*., bowels and stomach together, Supplementary Table S5). We assessed the degree of overlap between each of these labels and the morphological clusters to determine the association and impact that each had on tumor morphological phenotype. Additionally, we investigated the contribution of specific radiomic feature classes, shape (*n* = 14 features), intensity (*n* = 396 features), and texture (*n* = 1,606 features), by performing *t*-SNE and clustering on each class independently. Finally, we compared the degree of overlap between the clustering and endpoint labels using either features derived from the full lesion segmentation or those derived from an “eroded” segmentation, in which morphological binary erosion (using a 3D 6-connected structuring element) removed the peripheral tumor boundary, effectively shrinking each lesion by one voxel in all directions while preserving connectivity.

### Statistical analysis

The performance of agglomerative clustering and its concordance with ground-truth labels (tumor type and tumor environment) were assessed using two metrics. First, we calculated the Silhouette score, which ranges from -1 to +1 and evaluates intra-cluster cohesion *versus* inter-cluster separation [69]. Second, we used the adjusted Rand index (ARI) to quantify the overlap between predicted clusters and ground-truth classes [70]. Bayesian optimization used a combination of both methods with equal weights to optimize the clustering hyperparameters.

To determine the relative influence of tumor type *versus* tumor environment, separate ARI values for each factor were computed. We performed 100 bootstrap iterations to obtain a distribution of ARI values for both tumor type and environment, reporting the mean and 95% confidence intervals (CI). A one-sided bootstrap hypothesis test was conducted to evaluate statistical differences between the two groups, where a *p*-value below 0.05 was considered significant. Additionally, we generated contingency tables illustrating the distribution of lesions in each ground-truth category across the identified clusters. All analyses were performed in Python (v3.12.7) [71] using the following libraries: pandas (v2.2.2), numpy (v1.26.4), sklearn (v1.5.1), simpleitk (v2.4.1), hyperopt (v0.2.7), scipy (v1.13.1), matplotlib (v3.9.2).

## Results

### Patient characteristics

The discovery cohort included 1,598 cancer patients, almost evenly split between males (*n* = 780, 49%) and females (*n* = 818, 51%). The median age was 62 years (interquartile range 54‒69 years). After eligibility screening of the public datasets, the external validation cohort ultimately comprised 2,440 scans. Manual segmentation identified a total of 10,485 lesions in the discovery set and 6,597 lesions in the validation set.

Across both cohorts, 21 distinct primary tumor types were represented (*n* = 20 in the discovery set and *n* = 13 in the validation set). The most common tumor types in the discovery cohort were lung (*n* = 617, 39%), colorectal (*n* = 394, 25%), and sarcoma (*n* = 136, 9%). Comparably, the three most prevalent tumor types in the validation cohort were colorectal (*n* = 581, 24%), pancreas (*n* = 512, 21%), and lung tumors (*n* = 470, 19%).

A total of 14 anatomical environments were studied across all the lesions in both sets. In the discovery cohort, the three most common environments were lung (*n* = 2,785, 27%), lymph nodes (*n* = 2,599, 25%), and hepatobiliary system (*n* = 2,258, 22%). For the validation, the dominant environments were similar: lung (*n* = 2,079, 32%), hepatobiliary (*n* = 1,787, 27%), and lymph nodes (*n* = 1,471, 22%).

Regarding lesion type, the majority of segmented lesions in the discovery cohort were solid organ metastases (*n* = 7,052, 67%), followed by lymph node metastases (*n* = 2,598, 25%) and primary tumors (*n* = 835, 8%). In contrast, the validation cohort contained a relatively higher proportion of primary tumors, which made up the largest share of lesions (*n* = 2,747, 42%), followed by solid organ metastatic lesions (*n* = 2,380, 36%) and lymph node metastases (*n* = 1,470, 22%). A detailed breakdown of lesion types, tumor types, and anatomical environments is provided in Table 1. Tumor histological subtypes for the discovery cohort are listed in Supplementary Table S6.

**Table 1:** Detailed description of discovery and validation cohorts

Characteristic		Discovery				Validation			
		*N* (patients)	%	*N* (lesions)	%	*N* (patients)	%	*N* (lesions)	%
Overall		1,598	100.00	10,485	100.00	2,440	100.00	6,597	100.00
Lesion type	Primary	774	48.43	835	7.96	2,065	84.63	2,747	41.64
	Solid organ metastasis	1141	71.40	7,052	67.25	609	24.95	2,380	36.07
	Lymph node	710	44.43	2,598	24.77	485	19.87	1,470	22.28
Tumor type	Lung	617	38.61	4,351	41.49	470	19.26	1,915	29.02
	Colorectal	394	24.65	2,544	24.26	581	23.81	1,243	18.84
	Sarcoma	136	8.51	472	4.50	175	7.17	369	5.59
	Melanoma	88	5.50	401	3.82	107	4.38	657	9.95
	Ovary	48	3.00	319	3.04	57	2.33	312	4.72
	Breast	45	2.81	460	4.38	0	0.00	0	0.00
	Uterus and cervix	45	2.81	322	3.07	42	1.72	132	2.00
	Gastroesophageal	43	2.69	291	2.77	55	2.25	89	1.34
	Head and neck	36	2.25	215	2.05	0	0.00	0	0.00
	Bladder	22	1.37	173	1.65	78	3.19	89	1.34
	Appendix	19	1.18	62	0.59	0	0.00	0	0.00
	Renal	17	1.06	107	1.02	219	8.97	445	6.74
	Thymus	15	0.93	133	1.26	0	0.00	0	0.00
	Pancreas	14	0.87	154	1.46	512	20.98	955	14.47
	Small intestine	14	0.87	94	0.89	0	0.00	0	0.00
	Biliary	12	0.75	130	1.24	0	0.00	0	0.00
	Prostate	12	0.75	89	0.84	2	0.08	3	0.04
	Pleura	12	0.75	97	0.92	0	0.00	0	0.00
	Testicular	5	0.31	39	0.37	0	0.00	0	0.00
	Thyroid	4	0.25	32	0.30	6	0.24	10	0.15
	Liver	0	0.00	0	0.00	136	5.57	378	5.73
Tumor environment	Lung	743	46.49	2,785	26.56	599	24.54	2,079	31.51
	Lymph node	710	44.43	2,599	24.78	485	19.87	1,471	22.29
	Hepatobiliary	468	29.28	2,258	21.53	966	39.59	1,787	27.08
	Gastrointestinal	310	19.39	343	3.27	560	22.95	565	8.56
	Bone	255	15.95	997	9.50	19	0.77	40	0.60
	Peritoneum	232	14.51	896	8.54	55	2.25	146	2.21
	Adrenal	112	7.00	147	1.40	18	0.73	29	0.44
	Subcutaneous fat	58	3.63	167	1.59	7	0.28	9	0.13
	Gynecological	52	3.19	60	0.57	90	3.68	95	1.44
	Urinary system	38	2.44	51	0.48	295	12.09	349	5.29
	Head and neck	32	2.00	33	0.31	8	0.32	8	0.12
	Brain	12	0.75	38	0.36	0	0.00	0	0.00
	Breast	7	0.43	10	0.09	0	0.00	0	0.00
	Others	67	4.19	101	0.96	12	0.49	19	0.28

### Morphological embedding and clustering

**Fig. 1:**
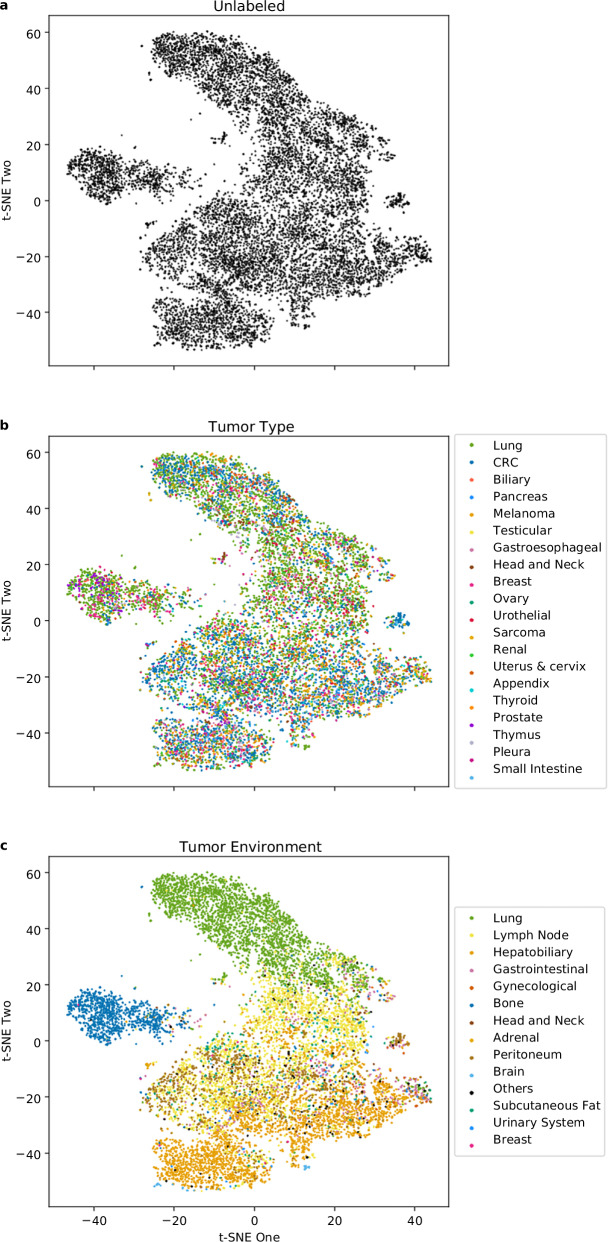
t-SNE morphological representation of the discovery cohort. **a** Unlabeled morphological representation of lesions. **b** Lesions labeled per tumor type. **c** Lesions labeled per tumor environment class

From each segmented lesion, we extracted 2,016 radiomic features that quantitatively describe its morphological phenotype. To visualize potential similarities and groupings within this high-dimensional feature space, we applied *t*-SNE for unsupervised dimensionality reduction. The resulting *t*-SNE maps of all lesions in the discovery cohort (*n* = 10,485) revealed multiple visually coherent morphological “islands” (Fig. 1a). When ground-truth labels were overlaid, these morphological patterns aligned much more closely with the anatomical environment than with tumor type (Fig. 1b, c).

**Fig. 2:**
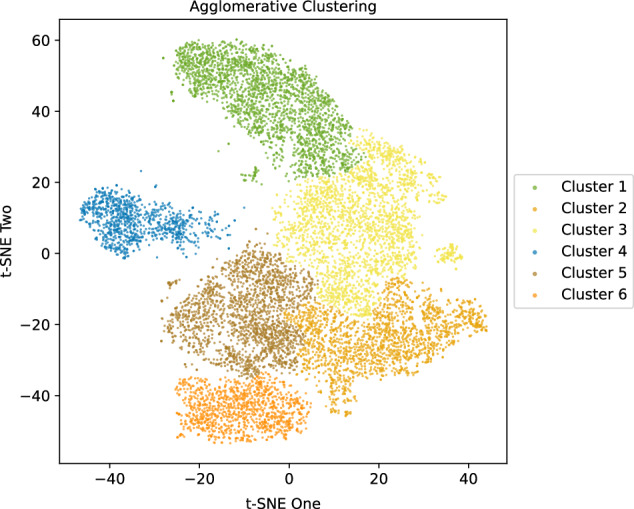
Agglomerative clustering in the discovery cohort. Morphologically similar clusters, identified using unsupervised agglomerative clustering in the discovery cohort, are shown

To quantify these visual observations, we performed agglomerative clustering on the *t*-SNE embeddings, which identified six morphologically distinct tumor clusters in the discovery cohort (Fig. 2), achieving a moderate Silhouette score of 0.44. Concordance analysis confirmed minimal overlap between these unsupervised clusters and the primary tumor type (mean ARI = 0.04, 95% CI: 0.02–0.06). In contrast, clustering showed substantially greater agreement with the anatomical environment (mean ARI = 0.37, 95% CI: 0.34–0.40). The mean difference in ARI between tumor environment and tumor type was 0.33 (95% CI: 0.29–0.36), with a one-sided bootstrap test confirming statistical significance (*p* < 0.010).

The tumor environment-driven results were further supported by the cluster–environment cross-distribution shown in Table 2. For instance, cluster 1 was predominantly composed of lung lesions (79%), while cluster 4 was almost entirely composed of bone metastases (93%). Other clusters similarly displayed clear enrichment for single or more dominant anatomical sites, reinforcing that the unsupervised morphological clusters mapped more closely to distinct tissue environments rather than primary tumor lineage.

**Table 2:** Percentage distribution of tumor environment classes across morphological clusters in the discovery cohort

Ground truth	Cluster 1	Cluster 2	Cluster 3	Cluster 4	Cluster 5	Cluster 6
Adrenal	0	42.18	5.44	0	46.26	6.12
Bone	0.6	0.9	3.61	92.68	2.01	0.2
Brain	0	34.21	15.79	0	2.63	47.37
Breast	0	10	20	10	40	20
Gastrointestinal	1.46	35.57	37.9	4.08	19.53	1.46
Gynecological	0	65	28.33	0	5	1.67
Head and neck	12.12	15.15	69.7	3.03	0	0
Hepatobiliary	0.13	41.54	8.64	0.18	9.7	39.81
Lung	79.17	1.9	17.27	0.25	1.36	0.04
Lymph node	2.62	11.81	45.67	0.88	35.05	3.96
Peritoneum	0.11	21.09	20.65	0.89	52.68	4.58
Subcutaneous fat	0	15.57	38.32	0	41.32	4.79
Urinary system	0	35.29	23.53	0	23.53	17.65
Others	1.98	40.59	29.7	3.96	17.82	5.94

**Fig. 3:**
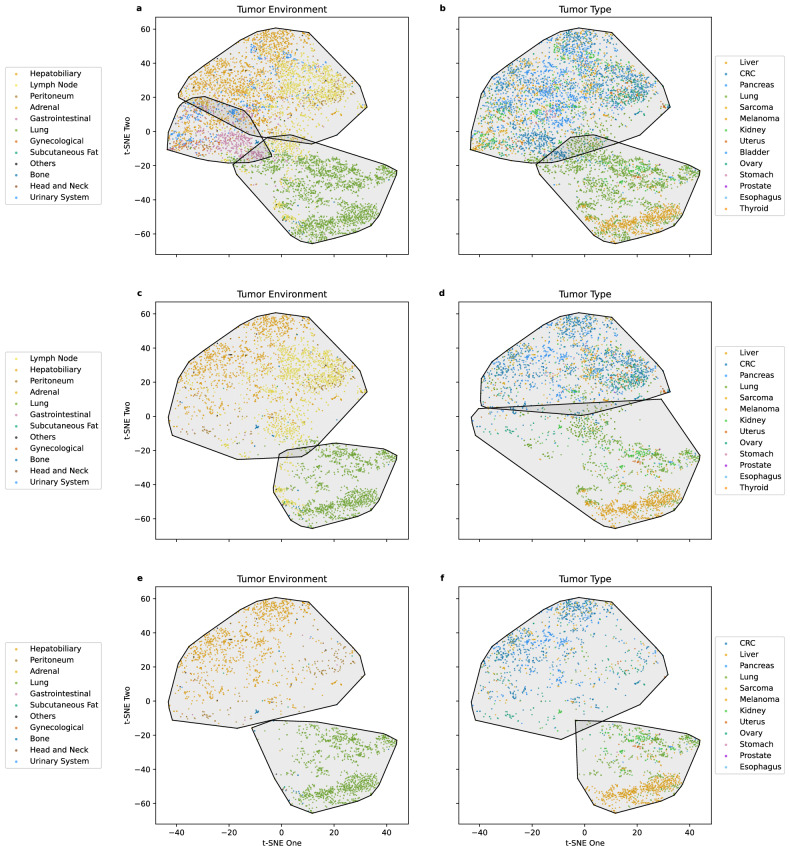
Agglomerative clustering in the validation cohort. **a** All lesions labeled by tumor environment and outlined per cluster. **b** All lesions labeled by tumor type and outlined per cluster. **c** Metastatic lesions labeled by tumor environment and outlined per cluster. **d** Metastatic lesions labeled by tumor type and outlined per cluster. **e** Solid organ metastatic lesions labeled by tumor environment and outlined per cluster. **f** Solid organ metastatic lesions labeled by tumor type and outlined per cluster

A similar phenomenon was observed in the external validation cohort (*n* = 6,597 lesions), where *t*-SNE again revealed distinct morphological groupings that tracked the local environment more than tumor type (Fig. 3a, b). Clustering in the validation set yielded a comparable Silhouette score of 0.42, with mean ARI values of 0.40 (95% CI: 0.32–0.48) for the environment *versus* 0.20 (95% CI: 0.14–0.26) for the tumor type (Table 3). The bootstrap test confirmed this difference remained significant (*p* < 0.010).

**Table 3:** Outcomes of clustering analysis in the discovery and validation cohorts

Analysis	Cohort	Ground truth	Mean ARI	95% CI (Mean ARI)	Original dataset ARI	Mean difference in ARI	95% CI (mean difference)	*p*-value
All lesions	Discovery (*n* = 10,485)	Tumor type	0.04	[0.02, 0.06]	0.05	0.33	[0.29, 0.36]	*p* < 0.01
		Tumor environment	0.37	[0.34, 0.40]	0.38			
	Validation (*n* = 6,597)	Tumor type	0.20	[0.14, 0.26]	0.18	0.20	[0.11, 0.31]	*p* < 0.01
		Tumor environment	0.40	[0.32, 0.48]	0.44			
Metastatic lesions	Discovery (*n* = 9,650)	Tumor type	0.03	[0.02, 0.04]	0.04	0.38	[0.34, 0.42]	*p* < 0.01
		Tumor environment	0.40	[0.38, 0.45]	0.41			
	Validation (*n* = 3,850)	Tumor type	0.20	[0.14, 0.24]	0.22	0.19	[0.13, 0.28]	*p* < 0.01
		Tumor environment	0.39	[0.36, 0.46]	0.37			
Solid organ metastatic lesions	Discovery (*n* = 7,052)	Tumor type	0.05	[0.04, 0.07]	0.05	0.54	[0.51, 0.59]	*p* < 0.01
		Tumor environment	0.60	[0.56, 0.64]	0.57			
	Validation (*n* = 2,380)	Tumor type	0.31	[0.29, 0.33]	0.31	0.49	[0.46, 0.51]	*p* < 0.01
		Tumor environment	0.80	[0.76, 0.82]	0.80			

### Robustness of environment dominance in metastatic and solid organ metastatic lesions

**Fig. 4:**
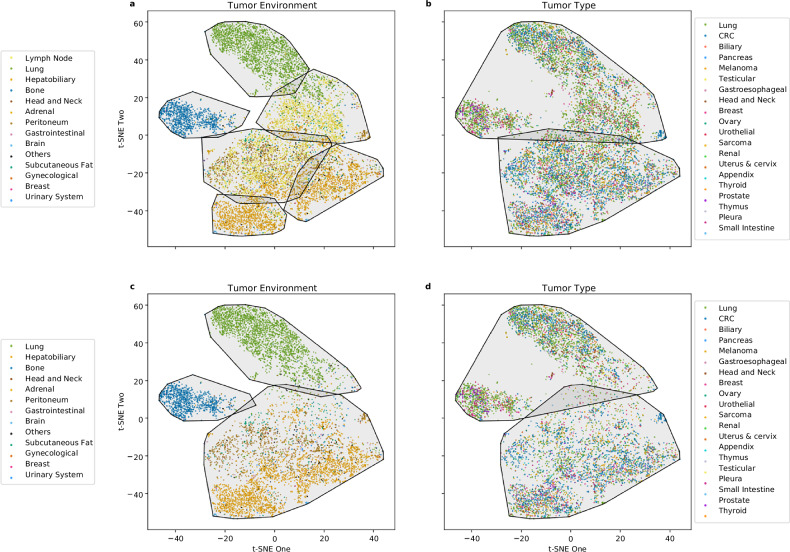
Agglomerative clustering in the discovery cohort of metastatic and solid organ metastatic lesions. **a** Metastatic lesions labeled by tumor environment and outlined per cluster. **b** Metastatic lesions labeled by tumor type and outlined per cluster. **c** Solid organ metastatic lesions labeled by tumor environment and outlined per cluster. **d** Solid organ metastatic lesions labeled by tumor type and outlined per cluster

Since tumor type and tumor environment labels are similar for primary tumors, and lymph node metastases can vary in anatomical location, we conducted two additional clustering analyses: one focused on metastatic lesions only (excluding primary tumors, Fig. 4a, b) and another exclusively on solid organ metastases (excluding both primary tumors and lymph node metastases, Fig. 4c, d) to further probe the relative influence of these biological factors.

After excluding primary tumors, 9,650 lesions remained for analysis in the discovery cohort. In this subset, tumor type showed a mean ARI of 0.03 (95% CI: 0.02–0.04), while tumor environment retained a stronger association (mean ARI = 0.40, 95% CI: 0.38–0.45). The mean difference in ARI was 0.38 (95% CI: 0.34–0.42), with a one-sided bootstrap test confirming its significance (*p* < 0.010).

When observing the distribution of lesions within the given clusters, a number of patterns emerge. As observed in Table 2, the environment signal remained clear, with clusters showing distinct preferences for dominant environments. For example, Cluster 1 was still dominated by lung metastases (85%), whereas Cluster 4 primarily consisted of bone lesions (93%), mirroring the organ-driven pattern observed overall. Table 4 illustrates the environment–cluster distribution for metastatic lesions.

**Table 4:** Percentage distribution of tumor environment classes across morphological clusters in the discovery cohort of metastatic lesions

Ground truth	Cluster 1	Cluster 2	Cluster 3	Cluster 4	Cluster 5	Cluster 6
Adrenal	0	63.27	29.93	0	6.12	0.68
Bone	0.6	3.01	0.9	92.58	0.2	2.71
Brain	0	5.26	34.21	0	47.37	13.16
Breast	0	25	25	25	25	0
Gastrointestinal	2.7	48.65	27.03	5.41	2.7	13.51
Gynecological	0	26.67	46.67	0	6.67	20
Head and neck	0	14.29	28.57	14.29	0	42.86
Hepatobiliary	0.13	16.71	38.04	0.18	39.65	5.29
Lung	84.63	2.16	0.59	0.25	0.04	12.31
Lymph node	2.19	49.27	7.39	0.81	3.93	36.41
Peritoneum	0.11	66.96	18.3	0.89	4.8	8.93
Subcutaneous fat	0	53.5	15.29	0	5.1	26.11
Urinary system	0	47.22	22.22	0	22.22	8.33
Others	1.08	34.41	36.56	4.3	7.53	16.13

In the validation cohort, a similar trend was evident (Fig. 3c, d). For metastatic lesions (*n* = 3,850), the mean ARI for tumor type was 0.20 (95% CI: 0.14–0.24), while the ARI for tumor environment was nearly double at 0.39 (95% CI: 0.36–0.46), with a mean difference of 0.19 (95% CI = 0.13–0.28; *p* < 0.010, Table 3).

When the analysis was further restricted to solid organ metastatic lesions (excluding lymph node metastases), the discovery cohort included 7,052 lesions. Here, the mean ARI for tumor type remained low at 0.05 (95% CI: 0.04–0.07), whereas the mean ARI for tumor environment increased markedly to 0.60 (95% CI: 0.56–0.64), yielding a mean difference of 0.54 (95% CI: 0.51–0.59; *p* < 0.01). Using the raw radiomic feature vector (instead of TSNE embeddings) yielded the same results: mean ARI = 0.42 (95% CI: 0.30–0.45) for tumor environment and mean ARI = 0.05 (95% CI: 0.02–0.06) for tumor type in the discovery cohort (*p* < 0.010).

Unlike Tables 2 and 4, where some clusters showed partial mixing, clusters of solid organ metastases often comprised a single dominant environment class (*e.g*., Cluster 2 contained > 95% of lung lesions), highlighting the robustness of the environment signal when lymph nodes and primaries were excluded (Table 5).

**Table 5:** Percentage distribution of tumor environment classes across morphological clusters in the discovery cohort of solid organ metastatic lesions

Ground truth	Cluster 1	Cluster 2	Cluster 3
Adrenal	100	0	0
Bone	4.81	2.51	92.68
Brain	100	0	0
Breast	75	0	25
Gastrointestinal	81.08	13.51	5.41
Gynecological	93.33	6.67	0
Head and neck	71.43	14.29	14.29
Hepatobiliary	99.15	0.67	0.18
Lung	4.5	95.25	0.25
Peritoneum	98.21	0.78	1
Subcutaneous fat	97.45	2.55	0
Urinary system	100	0	0
Others	86.02	9.68	4.3

Again, this trend held in the validation cohort (Table 3). For solid organ metastatic lesions (*n* = 2,380), the mean ARI for tumor type was 0.31 (95% CI: 0.29–0.33). In comparison, the ARI for tumor environment increased sharply to 0.80 (95% CI: 0.76–0.82), with a significant mean difference of 0.49 (95% CI: 0.46–0.51; *p* < 0.010) (Fig. 3e, f). The same was observed when using the raw radiomic feature vector for tumor environment (mean ARI = 0.75 (95% CI: 0.63–0.81)) and tumor type (mean ARI = 0.35 (95% CI: 0.29–0.39, *p* = 0.020)).

### Contribution of shape, intensity, and texture features to the morphological concordance with biological ground truth

**Fig. 5:**
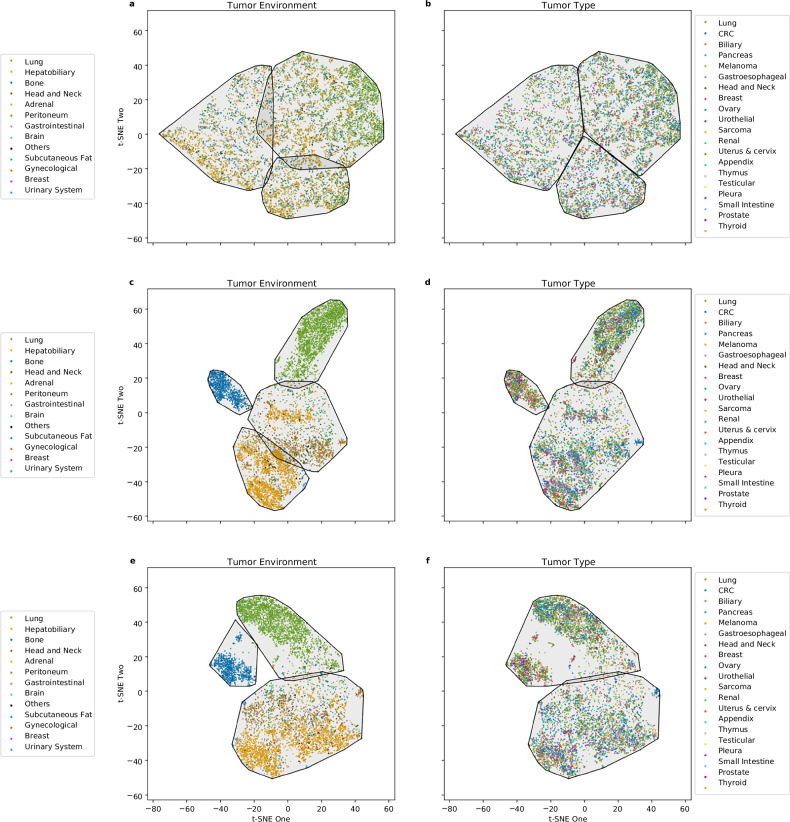
Agglomerative clustering in the discovery cohort of solid organ metastatic lesions with subsets of radiomic features. **a**, **b** Morphological representations based on shape features, labeled by tumor environment and tumor type, respectively. **c**, **d** Morphological representations based on intensity features, labeled by tumor environment and tumor type, respectively. **e**, **f** Morphological representations based on texture features, labeled by tumor environment and tumor type, respectively

Using the discovery cohort of solid organ metastatic lesions, we examined how different aspects of tumor morphology, specifically shape, intensity, and texture, independently relate to tumor type and anatomical environment. When restricting the analysis to shape-related radiomic features alone, the mean ARI was low for both biological factors: 0.02 (95% CI: 0.01–0.03) for tumor type and 0.06 (95% CI: 0.05–0.07) for tumor environment (Fig. 5a, b).

In contrast, intensity and texture features demonstrated markedly stronger associations with the tumor environment. Using only intensity-related features yielded a mean ARI of 0.02 (95% CI: 0.01–0.03) for tumor type but a substantially higher ARI of 0.64 (95% CI: 0.56–0.68) for the anatomical environment (Fig. 5c, d). Similarly, texture-only analysis resulted in a mean ARI of 0.05 (95% CI: 0.04–0.06) for tumor type and 0.56 (95% CI: 0.52–0.60) for environment (Fig. 5e, f).

### Impact of tumor core *versus* periphery on morphological concordance

To test whether peripheral tumor regions, which directly interface with surrounding tissue, predominantly drive the observed morphological clustering, we repeated the analysis using radiomic features extracted from eroded segmentations of solid organ metastatic tumors in the discovery cohort. This was achieved by removing one voxel from the periphery all around, while maintaining continuity (Supplementary Fig. S2). The results remained consistent: the mean ARI for tumor type was unchanged at 0.05 (95% CI: 0.04–0.08), while the mean ARI for tumor environment remained substantial at 0.50 (95% CI: 0.43–0.54). The mean difference in ARI was 0.44 (95% CI: 0.37–0.48), and a one-sided bootstrap test confirmed that the environmental effect remained significantly stronger than the lineage signal (*p* < 0.010), even after removing the peripheral tumor boundaries (Supplementary Fig. S3).

## Discussion

Our study demonstrates that tumors’ morphological phenotype on CT imaging is largely driven by a host tissue-related environmental “imprint” rather than by their site of origin. In a setting where all lesion types (primary tumors, solid organ metastases, and lymph node metastases) were studied, we observed a stronger association between unsupervised morphological clusters and the anatomical context of the tumors. As we focused more on the local environment, first by excluding primary tumors, and subsequently lymph node metastases, this association increased substantially. Notably, this relationship was maintained even after the periphery of the tumor segmentations was removed from the analysis, indicating that the signal originates more prominently from the tumor core rather than the transitional/peripheral areas.

Radiological research has long hinted at the influence of tissue context on tumor morphology. For instance, liver metastases from different primary sites often exhibit shared imaging features, such as peripheral rim washout/enhancement [72]. Similarly, pulmonary metastases tend to form round nodules, due to the low mechanical resistance of aerated lung tissue [73]. These observations, though well known to practicing radiologists, have remained constrained to semantic feature research. Our study quantitatively confirms this principle using high-dimensional radiomic analysis across multiple organs and tumor types.

In our two cohorts, the tumor morphological phenotype was not solely a product of its cell of origin but was also shaped by the local anatomical context in which it grew. When disparate cancers metastasize and grow in the same organ, they often converge toward a similar imaging appearance shaped by the local tissue environment. This observation mirrors two principles in cancer biology, namely the “seed and soil” hypothesis and plasticity. In the former, the hypothesis posits that the spread of metastases is not random but rather based on the suitability of the host environment for the disseminated cells. Upon arrival, metastatic cells adapt to the host environment to survive and proliferate [74, 75]. Likewise, plasticity refers to a phenomenon where tumor cells adapt to their new environment and modify their phenotype depending on the local microenvironmental needs [76]. Tumor plasticity has even been heralded as a potential new hallmark of cancer [77].

Modern transcriptomic studies support this idea, showing that metastases upregulate target organ-specific genes; for example, liver metastases activate hypoxia and angiogenesis pathways, while brain metastases express neural adhesion markers [78]. Hartung et al further demonstrated that disseminated tumor cells downregulate origin-specific genes and instead adopt transcriptional profiles more characteristic of the target organ [79].

Our imaging analysis can be understood as a radiological parallel of these biological phenomena. Just as metastases adjust their gene expression to reflect the destination organ (*e.g*., liver metastases adopting unique [80] or liver-like profiles), tumors may acquire morphological traits molded by the surrounding tissue architecture. This “environmental imprinting” suggests that radiomic features are shaped not only by tumor-intrinsic factors but also by the structural, molecular, and mechanical properties of the host organ. Factors such as stromal density, extracellular matrix composition, blood supply, mechanical stress, and motion (*e.g*., respiratory-induced blurring in the lung) likely contribute to these phenotypes.

These findings have eminent implications for understanding tumor heterogeneity: two lesions with identical molecular profiles may appear dramatically different on imaging if one grows in the lung and the other in bone, highlighting the spatial evolution of tumors where different environments drive divergent phenotypic trajectories [81]. These insights also have important translational indications, particularly in the development and deployment of AI models in oncology. There is growing enthusiasm for tissue-agnostic diagnostics and treatments; for example, the USA Food and Drug Administration approvals for neurotrophic tyrosine receptor kinase fusions or microsatellite instability-high tumors regardless of tumor origin [82]. However, our results suggest that while molecular biomarkers may transcend tissue of origin, tumor imaging phenotypes do not easily escape their local context. This is especially relevant (and challenging) for radiologists and AI model developers: a machine learning model trained to identify tumor *type* from imaging might be confounded if it doesn’t account for the organ environment.

In practical terms, a model might conflate spiculated margins with lung primaries, or smooth, encapsulated contours with liver metastases, not because these features are exclusive to those histologies, but because they are common morphological responses within those environments. This potential for misclassification can limit the generalizability of AI models across organ sites.

The study by Strotzer et al, the largest externally validated effort in this space, similarly found that radiomics alone was unable to reliably determine the primary tumor type of brain metastases, reinforcing the idea that metastases converge toward a common phenotype dictated by the destination organ [83]. Our findings echo this: while medical imaging analysis may not reliably identify tumor lineage in a tissue-agnostic fashion, it may robustly reflect the tumor’s anatomical context.

This limitation, however, may also present an opportunity. While radiomics may struggle to differentiate tumor types across organs, it appears highly effective at identifying the organ of implantation. It may be feasible to develop two-step AI models: first, determine the organ context with high accuracy, then apply specialized models trained to predict tumor type within that environment. This could be particularly valuable in diagnostically challenging cases such as cancers of unknown primary, where identifying the probable primary tumor site can guide biopsy and inform management decisions [84].

More broadly, our results invite a rethinking of conventional radiologic interpretation. Rather than expecting a consistent appearance for a given tumor type regardless of location, it may be more realistic to adopt a contextual phenotypic model in which tumor appearance is co-determined/shaped by lineage and local environment. Features such as spiculation, necrosis, and calcification patterns should be understood as products of the tumor-host interplay rather than intrinsic tumor properties. A deeper understanding of such interactions can improve diagnostic reasoning in complex cases, such as those involving atypical metastatic presentations or unusual primary tumors. Previous radiomics studies have largely focused on single-tumor-type cohorts, limiting their ability to uncover this broader principle. By aggregating a diverse set of tumors across multiple organs, we provide generalizable evidence that local environmental effects are a dominant force shaping tumor morphology.

While these results are encouraging, we acknowledge a number of limitations in our study. First, this is a retrospective radiomic analysis without direct correlation to molecular or clinical outcomes. The public external datasets lacked harmonized outcome and therapy annotations at scale, and post hoc analyses would risk confounding by indication and acquisition heterogeneity. Prospective studies with standardized clinical endpoints and integrated multi-omics are needed to test whether the identified morphology neighborhoods carry prognostic or predictive value. Moreover, we inferred tissue-based environmental influences from imaging patterns, but did not measure specific microenvironmental characteristics. As such, we cannot conclusively determine which specific microenvironmental factors (*e.g*., stromal fraction, immune infiltrate, or vascular density) are responsible for the observed morphological differences. Although we performed a robustness experiment that eroded the peripheral/transitional tumor boundary to focus on the core, we did not explicitly segment the peri-tumoral microenvironment (*e.g*., immediately adjacent stroma, microvasculature, and ductal/bronchiolar or ductal structures). Future work could extract dedicated peri-tumoral features (via ring-based or multi-compartment (tumor-plus-environment) segmentations) to examine these fine subcomponents more directly. Furthermore, our labels for anatomical locations were based on grouping organs/systems together. While necessary in our analyses to obtain sufficient samples per class, future dedicated studies can explore detailed organ-by-organ differences/associations. Second, there is the potential for confounding due to differences in imaging techniques. Organs are often imaged using protocol-specific parameters, especially in locoregional studies, which can introduce variability. To mitigate this, we restricted our dataset to contrast-enhanced, multi-regional thoraco-abdominopelvic scans acquired in the portal venous phase to help ensure consistency in image acquisition. However, residual differences in contrast administration and timing across centers (such as iodine concentration and volume, injection rate, bolus triggering/delay, and patient hemodynamics) may affect organ-specific enhancement and derived intensity/texture features. In the validation set, images were compiled from publicly available datasets where such parameters could not be controlled. We also did not apply explicit cross-scanner/protocol harmonization. Although images were resampled to isotropic voxels, features were standardized, and extraction followed Image Biomarker Standardization Initiative‒IBSI-compliant settings, residual vendor/protocol “batch” effects may persist. Future work should assess harmonization approaches (*e.g*., ComBat/Removal of artificial voxel effect by linear regression‒RAVEL, histogram standardization, domain-adversarial/domain-invariant models) and center-aware validation designs (*e.g*., leave-center/manufacturer-out) to further disentangle biological environment from acquisition effects. That being said, our observations held true even in such a heterogeneous cohort. Lastly, manual lesion segmentations introduce inter-reader variability. To address this, we followed standardized segmentation protocols and implemented quality control procedures. Formal quantification of inter- and intra-reader reproducibility (*e.g*., on repeat segmentations/test–retest cases), alongside evaluation of semi-automatic or deep-learning–assisted segmentation, could further improve scalability and consistency. However, the consistency of our findings across both the discovery and validation sets, as well as the robustness analysis, with the erosion of the segmentations, strengthens their generalizability.

In summary, our findings demonstrate that a tumor’s radiomic appearance is not solely determined by its biological origin but is influenced by the anatomical environment in which it resides. The observed silhouette values indicated moderate cluster cohesion. Accordingly, we interpret these clusters as coarse morphology neighborhoods rather than sharply separated phenotypes. This challenges the traditional notion of a fixed imaging phenotype per tumor type and supports a more nuanced, context-dependent model of tumor morphology. By quantifying how the local environment shapes radiologic features, our study lays the groundwork for more biologically informed interpretation of imaging and for developing AI models that are both anatomically aware and clinically robust. As radiology moves toward precision oncology, recognizing and incorporating these context-driven effects will be essential for accurate diagnosis, classification, and treatment guidance.

## Supplementary information

**Additional file 1**: **Supplementary Figure S1**. Consort flow diagram, depicting the inclusion process in the discovery cohort. **Supplementary Figure S2**. Example of original and eroded segmentations. Contrast-enhanced CT scan (portal venous phase with soft tissue windowing) showing a liver metastasis in a colorectal cancer patient from the discovery cohort. In the yellow outline, the original segmentation is shown, which is meant to cover the full lesion. In solid red, an eroded segmentation is shown, with one voxel systematically removed from the periphery. **Supplementary Figure S3**. Clustering analysis exclusively on central tumor regions. (**A**) Lesions labeled by tumor environment and outlined per cluster. (**B**) Lesions labeled by tumor type and outlined per cluster. **Supplementary Table S1**. Included publicly available datasets within the validation cohort. **Supplementary Table S2**. Acquisition parameters for the discovery and validation sets. Continuous variables are reported as median values with interquartile ranges, while categorical variables are presented as absolute counts and corresponding percentages. Model names occurring in less than 1% of their respective datasets are grouped under the category “Other.”

## Data Availability

The datasets generated and/or analyzed during the current study are not publicly available due to national and institutional patient privacy regulations, but are available from the corresponding author upon reasonable request. Public datasets used in this research are available directly from their respective sources.

## Abbreviations

AI: Artificial intelligence
ARI: Adjusted Rand index
CI: Confidence interval
CT: Computed tomography
PACS: Picture Archiving and Communication System
*t*-SNE: *t*-distributed stochastic neighbor embedding

## Code availability

The code used to perform the experiments is accessible at https://github.com/nki-radiology/Tumour_morphology_environment_vs_tumour_type.
