# Supplementary material for: Tumor morphology on CT radiomics is largely driven by the local anatomical environment, not the primary tumor type
Source: Eur Radiol Exp. 2026 Mar 12;10:26. doi: 10.1186/s41747-026-00691-5 (PMC12982741; doi:10.1186/s41747-026-00691-5)
Supplement: Supplementary file 1 — Additional file 1: Supplementary Figure S1. Consort flow diagram, depicting the inclusion process in the discovery cohort. Supplementary Figure S2. Example of original and eroded segmentations. Contrast-enhanced CT scan (portal venous phase with soft tissue windowing) showing a liver metastasis in a colorectal cancer patient from the discovery cohort. In the yellow outline, the original segmentation is shown, which is meant to cover the full lesion. In solid red, an eroded segmentation is shown, with one voxel systematically removed from the periphery. Supplementary Figure S3. Clustering analysis exclusively on central tumor regions. (A) Lesions labeled by tumor environment and outlined per cluster. (B) Lesions labeled by tumor type and outlined per cluster. Supplementary Table S1. Included publicly available datasets within the validation cohort. Supplementary Table S2. Acquisition parameters for the discovery and validation sets. Continuous variables are reported as median values with interquartile ranges, while categorical variables are presented as absolute counts and corresponding percentages. Model names occurring in less than 1% of their respective datasets are grouped under the category “Other.” [file 41747_2026_691_MOESM1_ESM.pdf]

# Tumor Morphology on CT Radiomics is Largely Driven by the Local Anatomical Environment, Not the Primary Tumor Type

## ELECTRONIC SUPPLEMENTARY MATERIAL

**Supplementary Figure S1. Consort flow diagram;** depicting the inclusion process in the discovery cohort.

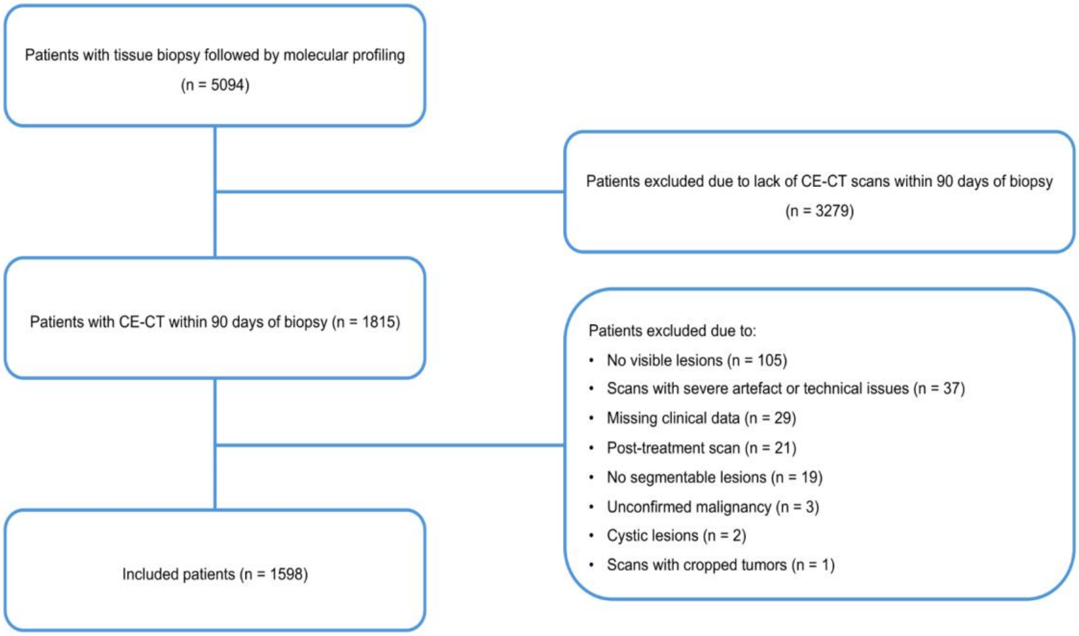

**Supplementary Figure S2. Example of original and eroded segmentations.** Contrast-enhanced CT scan (portal venous phase with soft tissue windowing) showing a liver metastasis in a colorectal cancer patient from the discovery cohort. In the yellow outline, the original segmentation is shown that is meant to cover the full lesion. In solid red, an eroded segmentation is shown, with one voxel systematically removed from the periphery.

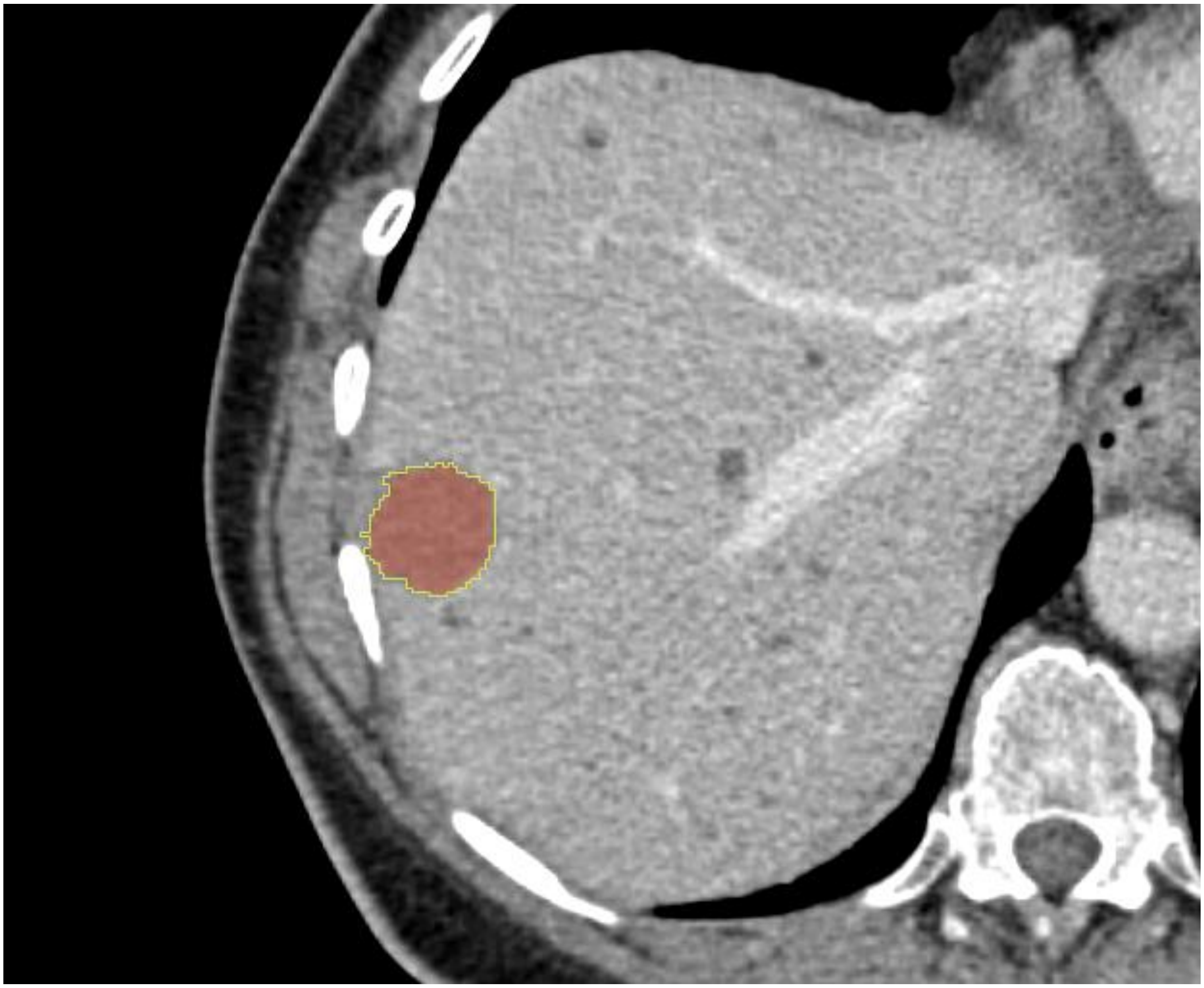

**Supplementary Figure S3. Clustering analysis exclusively on central tumor regions.** (A) Lesions labeled by tumor environment and outlined per cluster. (B) Lesions labeled by tumor type and outlined per cluster.

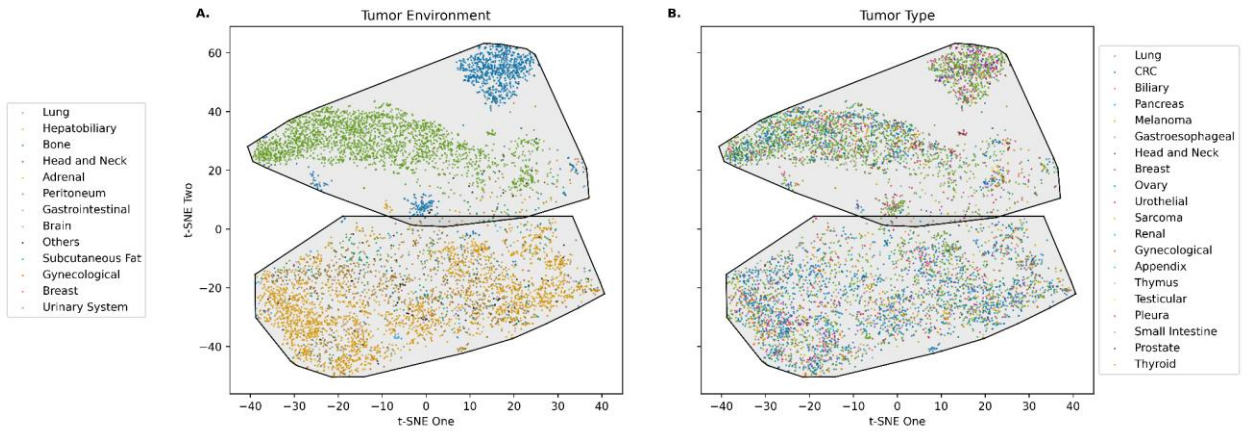

**Supplementary Table S1. Included publicly available datasets within the validation cohort.**

| Publicly available dataset | Number of patients | Number of included patients |
|---|---|---|
| Task07_Pancreas | 421 | 405 |
| WORC-GIST | 246 | 166 |
| CRLM-CT | 197 | 165 |
| StageII-Colorectal-CT | 230 | 156 |
| TCGA-KIRC | 267 | 154 |
| Task10_Colon | 190 | 140 |
| HCC-TACE-Seg | 105 | 99 |
| WORC-Melanoma | 103 | 98 |
| DEC_Lung | 96 | 94 |
| Lung-PET-CT-Dx | 355 | 94 |
| TCGA-BLCA | 120 | 78 |
| WORC-CRLM | 77 | 66 |
| CPTAC-PDA | 107 | 64 |
| LungCT-Diagnosis | 61 | 59 |
| TCGA-OV | 143 | 57 |
| CPTAC-CCRCC | 60 | 41 |
| TCGA-STAD | 46 | 41 |
| QIN LUNG | 47 | 40 |
| TCGA-LIHC | 97 | 37 |
| panNET | 76 | 37 |
| TCGA-LUAD | 69 | 36 |
| Anti-PD-1_Lung | 86 | 29 |
| Task09_Spleen | 61 | 27 |
| CPTAC-LSCC | 36 | 25 |
| TCGA-UCEC | 65 | 23 |
| TCGA-LUSC | 37 | 22 |
| NSCLC-Radiomics-Interobserver1 | 22 | 22 |
| RIDER Lung CT | 56 | 21 |

| | | |
|---|---|---|
| CPTAC-UCEC | 72 | 19 |
| TCGA-COAD | 25 | 15 |
| CPTAC-LUAD | 25 | 13 |
| TCGA-ESCA | 16 | 13 |
| TCGA-KICH | 15 | 12 |
| TCGA-KIRP | 33 | 12 |
| CMB-CRC | 12 | 10 |
| CMB-LCA | 10 | 8 |
| CPTAC-SAR | 24 | 8 |
| RIDER Pilot | 32 | 7 |
| Pancreatic-CT-CBCT-SEG | 40 | 6 |
| TCGA-THCA | 6 | 6 |
| CPTAC-CM | 13 | 5 |
| CMB-MEL | 9 | 4 |
| TCGA-READ | 3 | 2 |
| CMB-PCA | 3 | 1 |
| TCGA-PRAD | 14 | 1 |
| TCGA-SARC | 3 | 1 |
| CMB-GEC | 1 | 1 |

**Supplementary Table S2. Acquisition parameters for the discovery and validation sets.** Continuous variables are reported as median values with interquartile ranges, while categorical variables are presented as absolute counts and corresponding percentages. Model names occurring in less than 1% of their respective datasets are grouped under the category "Other."

| Parameter | Discovery set | | Validation set | |
|---|---|---|---|---|
| Slice Thickness [mm] | 1.0 (1.0–1.5) | | 4.0 (2.5–5.0) | |
| Pixel Spacing [mm] | 0.8 (0.7–0.8) | | 0.8 (0.7–0.8) | |
| kVp [kV] | 120.0 (120.0–120.0) | | 120.0 (120.0–120.0) | |
| Tube Current [mAs] | 180.5 (138.0–294.0) | | 265.0 (170.0–360.0) | |
| Exposure [mAs] | 96.0 (72.0–168.0) | | 106.0 (28.0–217.5) | |
| Model | Canon Aquilion | 898 (56.2%) | GE LightSpeed16 | 234 (15.1%) |
| | Siemens SOMATOM Sensation Open | 389 (24.3%) | Siemens SOMATOM Sensation 64 | 209 (13.5%) |
| | Siemens SOMATOM Confidence | 146 (9.1%) | GE LightSpeed VCT | 124 (8.0%) |
| | Philips GEMINI TF TOF 16 | 62 (3.9%) | Siemens SOMATOM Sensation 16 | 115 (7.4%) |
| | Philips Vereos PET/CT | 24 (1.5%) | Siemens SOMATOM Sensation 40 | 56 (3.6%) |
| | Other | 79 (4.9%) | GE Discovery CT750 HD | 48 (3.1%) |
| | | | GE LightSpeed QX/i | 47 (3.0%) |
| | | | Canon Aquilion | 47 (3.0%) |
| | | | Philips Brilliance 64 | 43 (2.8%) |
| | | | GE LightSpeed Ultra | 37 (2.4%) |
| | | | Siemens SOMATOM Volume Zoom | 32 (2.1%) |
| | | | GE LightSpeed Plus | 25 (1.6%) |
| | | | GE BrightSpeed | 24 (1.5%) |

| | | | | |
|---|---|---|---|---|
| | | | Siemens Emotion 6 | 19 (1.2%) |
| | | | Other | 306 (19.7%) |
| | | | Missing | 184 (11.8%) |

**Supplementary Table S3. t-SNE hyperparameters.**

| Parameter | Value |
|---|---|
| **n_components** | 2 |
| **perplexity** | Square root of num_lesions |
| **early_exaggeration** | 12.0 |
| **learning_rate** | auto |
| **max_iter** | 1000 |
| **n_iter_without_progress** | 300 |
| **min_grad_norm** | 1e-7 |
| **metric** | euclidean |
| **metric_params** | None |
| **init** | random |
| **verbose** | 0 |
| **random_state** | 42 |
| **method** | barnes_hut |
| **angle** | 0.5 |
| **n_jobs** | -1 |

**Supplementary Table S4. Optimized clustering hyperparameters.**

| Experiment | | | | Hyperparameters | | |
|---|---|---|---|---|---|---|
| **Cohort** | **Features** | **Lesions** | **Label** | **n_clusters** | **linkage** | **affinity** |
| **Discovery** | **All** | **All** | **Tumor environment** | 6 | ward | euclidean |
| **Discovery** | **All** | **All** | **Tumor type** | 2 | average | cosine |
| **Discovery** | **All** | **Metastatic** | **Tumor environment** | 6 | average | manhattan |
| **Discovery** | **All** | **Metastatic** | **Tumor type** | 2 | average | manhattan |
| **Discovery** | **All** | **Solid metastatic** | **Tumor environment** | 3 | ward | euclidean |
| **Discovery** | **All** | **Solid metastatic** | **Tumor type** | 2 | ward | euclidean |
| **Discovery** | **Shape** | **Solid metastatic** | **Tumor environment** | 3 | ward | euclidean |
| **Discovery** | **Shape** | **Solid metastatic** | **Tumor type** | 3 | average | cosine |
| **Discovery** | **Intensity** | **Solid metastatic** | **Tumor environment** | 4 | complete | l1 |
| **Discovery** | **Intensity** | **Solid metastatic** | **Tumor type** | 3 | complete | l1 |
| **Discovery** | **Texture** | **Solid metastatic** | **Tumor environment** | 3 | average | l1 |
| **Discovery** | **Texture** | **Solid metastatic** | **Tumor type** | 2 | average | l1 |
| **Discovery (eroded)** | **All** | **Solid metastatic** | **Tumor environment** | 2 | average | cosine |
| **Discovery (eroded)** | **All** | **Solid metastatic** | **Tumor type** | 2 | average | cosine |
| **Validation** | **All** | **All** | **Tumor environment** | 3 | average | l1 |

| | | | | | | |
|---|---|---|---|---|---|---|
| **Validation** | **All** | **All** | **Tumor type** | 2 | average | l1 |
| **Validation** | **All** | **Metastatic** | **Tumor environment** | 2 | average | l2 |
| **Validation** | **All** | **Metastatic** | **Tumor type** | 2 | complete | cosine |
| **Validation** | **All** | **Solid metastatic** | **Tumor environment** | 2 | complete | euclidean |
| **Validation** | **All** | **Solid metastatic** | **Tumor type** | 2 | average | l1 |

**Supplementary Table S5. Anatomical site label categories.** The table lists the specific anatomical sites included within each label category.

| Label | Included anatomical sites |
|---|---|
| **Lung** | Lung, Lower airways, Pleura |
| **Lymph node** | Lymph nodes |
| **Hepatobiliary** | Liver, Pancreas, Gall bladder, and bile ducts |
| **Gastrointestinal** | Small intestine, Colon and rectum, Stomach, Esophagus |
| **Bone** | Bone |
| **Peritoneum** | Peritoneum |
| **Adrenal** | Adrenal gland |
| **Subcutaneous fat** | Subcutaneous fat |
| **Gynecological** | Ovary, Uterus, Cervix, and genitals |
| **Urinary system** | Kidney, Bladder, Prostate |
| **Head and neck** | Thyroid, Thymus, Parotid gland, and other head & neck tumors |
| **Brain** | Brain |
| **Breast** | Breast |
| **Others** | Heart, Spleen, Skin, Muscle, and unspecified mediastinal lesions |

**Supplementary Table S6. Histological subtypes in the discovery cohort.** Number and percentage of each histological subtype are provided.

| Label | Count (%) |
|---|---|
| **Adenocarcinoma** | 868 (54.31 %) |
| **Squamous cell carcinoma** | 127 (7.94 %) |
| **Gastrointestinal stromal tumor** | 108 (6.75 %) |
| **Carcinoma, NOS** | 70 (4.38 %) |
| **Melanoma** | 56 (3.50 %) |
| **Non -small cell carcinoma** | 43 (2.69 %) |
| **Ductal and Lobular carcinoma** | 40 (2.50 %) |
| **Neuroendocrine carcinoma** | 26 (1.62 %) |
| **Neuroendocrine tumor** | 26 (1.62 %) |
| **Large cell carcinoma** | 25 (1.56 %) |
| **Sarcoma** | 22 (1.37 %) |
| **Urothelial carcinoma** | 20 (1.25 %) |
| **Small cell carcinoma** | 13 (0.81 %) |
| **Acinar cell carcinoma** | 12 (0.75 %) |
| **Mesothelioma** | 10 (0.62 %) |
| **Basal cell carcinoma** | 9 (0.56 %) |
| **Thymoma** | 6 (0.37 %) |
| **Merkel cell carcinoma** | 5 (0.31 %) |
| **Renal cell carcinoma** | 4 (0.25 %) |
| **Medullary carcinoma** | 3 (0.18 %) |
| **Thymic carcinoma** | 2 (0.12 %) |
| **Cholangiocarcinoma** | 2 (0.12 %) |
| **Germ cell tumor** | 2 (0.12 %) |
| **Others** | 22 (1.37 %) |
| **Not available** | 77 (4.81 %) |

**Supplementary Methods S1. Radiomic feature extraction parameters.** The parameters below were used to define the feature extraction on PyRadiomics. “ImageType” refers to the filters applied to the different classes of features in “featureClass”. A custom bin width was selected per filter based on the value of the range. Further information on the parameters can be found in the PyRadiomics documentation.

```
imageType:
  Original:
    binWidth: 6
  Square:
    binWidth: 4
  SquareRoot:
    binWidth: 10
  Logarithm:
    binWidth: 10
  Exponential:
    binWidth: 1
  Gradient:
    binWidth: 5
  LoG (Laplacian of Gaussian):
```

```
    binWidth: 4
    sigma: [1.0, 2.0, 3.0, 4.0, 5.0]        Wavelet: {}
  LBP3D:
    binWidth: 1.0

featureClass:
  shape:
  firstorder:
  glcm:
    - 'Autocorrelation'
    - 'JointAverage'
    - 'ClusterProminence'
    - 'ClusterShade'
    - 'ClusterTendency'
    - 'Contrast'
    - 'Correlation'
    - 'DifferenceAverage'
    - 'DifferenceEntropy'
```

```
    - 'DifferenceVariance'
    - 'JointEnergy'
    - 'JointEntropy'
    - 'Imc1'
    - 'Imc2'
    - 'Idm'
    - 'Idmn'
    - 'Id'
    - 'Idn'
    - 'InverseVariance'
    - 'MaximumProbability'
    - 'SumEntropy'
    - 'SumSquares'
  glrlm:
  glszm:
  gldm:
  ngtdm:
```

```yaml
setting:
  interpolator: 'sitkBSpline'
  resampledPixelSpacing: [1, 1, 1]
  padDistance: 10

  minimumROIDimensions: 2
  minimumROISize: 1
  correctMask: true

  voxelArrayShift: 1000

  label: 1
```
